# Chloroform Liniment for Dental Purposes

**Published:** 1860-10

**Authors:** 


*Chloroform Liniment for Dental Purposes.*—Take the white of an egg and add an equal bulk of chloroform, let it digest four hours at an ordinary temperature, when it is fit for use. Apply to the gum, immediately above the aching tooth, by saturating a piece of lint, one-half inch in length by one-fourth in width, upon this place a second piece of dry lint larger than the first, to protect the lips, which must be allowed to carefully fall upon the two, so as they may retain their place. We have found this an invaluable relief for such disturbances as are not dependent upon fully developed inflammation, such as neuralgic disturbances and the first stages of periostitis.

A more powerful liniment can be made by taking one part of white of egg and four parts of chloroform, put them in a bottle and plunge it completely in a water bath at the temperature of from 120° to 140° Fahrenheit. Gelatinization takes place in four minutes.

This preparation is to be rubbed on any painful part, when great relief will be experienced. The slow cauterization it produces, and the protection of the part from the atmosphere, render it a very powerful counter-irritant, as applied to the gums in the manner described; we have found it in many cases highly serviceable and recommend it to more general use.

The cold preparation is perhaps the best and easiest made, and when a decided effort is required, it should be repeatedly used. B.

